# Evaluation of immunodiagnostic tests for human gnathostomiasis using different antigen preparations of *Gnathostoma spinigerum* larvae against IgE, IgM, IgG, IgG1‐4 and IgG1 patterns of post‐treated patients

**DOI:** 10.1111/tmi.13679

**Published:** 2021-09-21

**Authors:** Issariya Ieamsuwan, Dorn Watthanakulpanich, Urai Chaisri, Poom Adisakwattana, Paron Dekumyoy

**Affiliations:** ^1^ Department of Helminthology Faculty of Tropical Medicine Mahidol University Bangkok Thailand; ^2^ Department of Tropical Pathology Faculty of Tropical Medicine Mahidol University Bangkok Thailand

**Keywords:** albendazole, human gnathostomiasis, IgG1‐ELISA, ivermectin, partially purified antigen

## Abstract

**Objectives:**

The aims of the study were two‐fold: (1) antigen (Ag) preparation and evaluation of three antigens of *Gnathostoma spinigerum* infective larvae (GsL3), crude somatic antigen (CSAg), excretory‐secretory antigen (ESAg) and partially purified antigens (namely P1Ag, P2Ag and P3Ag) to differentiate IgE, IgG, IgG1–4 and IgM for human gnathostomiasis diagnosis; and (2) application of the selected ELISA for following up stored sera of patients treated with ivermectin (IVM) and albendazole (ABZ).

**Methods:**

Different antigens were analysed by antibodies of gnathostomiasis cases, other parasite infections and healthy controls using indirect ELISA to differentiate IgE, IgG, IgG1–4 and IgM. Then, prominent antigen and immunoglobulin were used in antibody predictions of gnathostomiasis cases treated with albendazole or ivermectin.

**Results:**

Sensitivity of all evaluated ELISAs: IgM‐, IgG‐, IgG1‐ and IgG4‐ELISA, was 100%. IgM‐ELISA with CSAg and P3Ag exhibited the highest specificity of 99%. IgG‐ELISA with P2Ag resulted in the highest specificity of 92.3%. IgG1‐ELISA with P2Ag and P3Ag showed excellent results with 100% specificity. Finally, P2Ag evaluated IgG1 of the followed‐up cases with ABZ and IVM. Decreasing antibody IgG1 levels were mostly found in both treatments at Month 9 and long follow‐up was over 12 months. A *Gnathostoma* worm was extracted from each two treated patients.

**Conclusions:**

Using IgG1‐ELISA against P2Ag and P3Ag gave excellent results with 100% sensitivity and specificity. These tests can be an alternative to immunoblotting for gnathostomiasis. IgG1 decreased at least 9 months in most cases, so long‐term treatment should be performed over 1 year.

## INTRODUCTION

Human gnathostomiasis is mostly found in the tropical and subtropical regions. The *Gnathostoma* larva migrates through the human body, causing cutaneous gnathostomiasis or visceral larva migrans. Since the larvae cannot develop into adults in humans [1, 2], the detection of antibodies against *Gnathostoma* worm infection is routinely applied. IgG and IgG1‐4 are preferred in studies for detecting gnathostomiasis and, based on ELISA, crude somatic antigen (CSAg) and purified antigens of *G*. *spinigerum* larvae (GsL3) are employed, although conflicting results have been obtained. As known, the IgG to purified antigens showed excellent results, but CSAg always caused low sensitivity and specificity. For example, IgG‐ELISA using CSAg of GsL3 showed 87% sensitivity and 96.7% specificity [3] and 95% sensitivity, and only 8% specificity [4]. Among subclasses, IgG1‐ELISA to CSAg had the highest sensitivity (98%) while IgG2‐ELISA had the highest specificity (88%); both IgG1 for sensitivity and IgG2 for specificity are used in the diagnosis of gnathostomiasis [4]. However, a good result of ELISA is based on the selection of appropriate test conditions and also the selected samples of different diseases. In IgG‐immunoblot, 24 kDa GsL3 was a potential diagnostic antigen to all proven gnathostomiasis cases and showed no cross‐reaction with 16 parasitoses, except one false positive of paragonimiasis [5]. In contrast, IgG to 24 kDa Ag showed high sensitivity (91.6%) and moderate specificity (87.8%). However, IgG1‐4 showed lower sensitivities than IgG, but specificity of IgG4 detection was increased to 93.9% [6]. A recombinant antigen, rGslic18, showed 93.75% sensitivity and 97.01% specificity with an immunochromatography test (ICT), which was determined using 14 parasitoses [7]. To improve the efficiency of immunodiagnosis, partially purified and purified CSAg were produced as examples; 24 kDa GsL3Ag purified by anion exchange column chromatography showed a promising result of IgG‐ELISA for 17 parasitoses of 100% sensitivity and specificity [8]. In contrast, the lactose affinity‐purified protein antigens from CSAg of *G*. *binucleatum* larvae cross‐reacted with 7 of 8 sera of 5 parasitoses using IgG‐ELISA, but IgG‐immunoelectrotransfer showed no cross‐reaction. Both methods provided 100% sensitivity with 11 sera of gnathostomiasis binucleatum [9]. According to previous studies, no specific IgG and IgG subclasses can be used in a stand‐alone manner in the ELISA using CSAg, partially purified and purified antigens to diagnose gnathostomiasis. Therefore, the aims of this study were two‐fold: (1) antigen preparation and evaluation of three antigens of GsL3, crude somatic antigen (CSAg), excretory‐secretory antigen (ESAg) and partially purified antigens, namely P1Ag, P2Ag and P3Ag, to differentiate IgE, IgG, IgG1‐4 and IgM for gnathostomiasis diagnosis; and (2) application of the selected ELISA for following up stored sera of patients treated with ivermectin (IVM) and albendazole (ABZ).

## METHODS

### Sera

All stored sera were permitted to be used by the Immunodiagnostic Unit for Helminthic Infections, the Department of Helminthology, Faculty of Tropical Medicine, and approved by the Ethics Committee of the Faculty of Tropical Medicine, Mahidol University, Bangkok, Thailand (No. TMEC 14‐067). Thirty gnathostomiasis (homologous) sera were positive by worm recovery or immunoblotting test [5]. Among other infections (heterologous), 179 sera from 25 parasitoses and 30 healthy control sera (Table 1), and the follow‐up sera from gnathostomiasis patients were included. All sera and follow‐up samples were kept in a ‐80°C deep freezer at the Department.

**TABLE 1:** List of serum samples divided into three categories of infection: homologous, heterologous and healthy control. The status of those sera was proven by detection of developmental stages of parasites, adult worms and sero‐tests

Group	Diseases	Number	Diagnostic detections
1	Gnathostomiasis	30	Worms, immunoblot
2	Strongyloidiasis	10	Larvae
	Hookworm infection	10	Eggs, larvae
	Ascariasis	8	Eggs and worms
	Trichuriasis	10	Eggs and worms
	Enterobiasis	1	Eggs
	Toxocariasis	5	Immunoblot
	Trichinellosis	10	Muscle larvae, immunoblot
	Capillariasis	3	Eggs, adult worms
	Angiostrongyliasis	15	Worms, immunoblot
	Bancroftian filariasis	10	Microfilariae
	Brugian filariasis	10	Microfilariae
	Neurocysticercosis	10	Cyst, immunoblot
	Sparganosis	3	Sparganum
	Taeniasis	10	Eggs, scolex, and/or segments
	Hymenolepiasis nana	5	Eggs
	Opisthorchiasis	10	Adult worms
	Paragonimiasis heterotremus	10	Eggs and worms
	Fascioliasis	5	Eggs, immunoblot
	Minute intestinal fluke infections	10	Adult worms
	Creeping eruption	3	Clinical symptoms, negative for strongyloidiasis and gnathostomiasis
	Entamoebiasis	3	Cysts
	Giardiasis	3	Cysts
	*Blastocystis homonis* infection	5	Cysts
	Toxoplasmosis	5	IgG‐dye test
	*Plasmodium falciparum* malaria	5	Blood stages
3	Healthy sera	30	Negative by stool examinations, gnathostomiasis using immunoblot and other helminthic infections using ELISA and ten different helminth antigens

### Follow‐up sera

Thirty‐five gnathostomiasis patients with symptoms and signs of migratory swelling, local swelling, history of fish consumption, positive 24 kDa immunoblot and GsL3 recovery after treatments with ABZ and IVM were selected from the records. Occasionally, worms migrating near skin surfaces were found in ABZ‐ or IVM‐treated patients. Experienced physicians would extract those worms by needle or biopsy. Stored sera were divided as follows: group 1, 23 patients treated with 400 mg of albendazole twice daily for 21 days; and group 2, 12 patients treated with ivermectin at 0.2 mg/kg as a single dose. Before treatments, each patient's serum was collected as Month 0 (M 0), and the treatments with ABZ or IVM sera were collected every 3 months of post‐treatment (mpt) called M3, M6, M9, M12 and others. The antibody patern of post‐treatments was followed up until regularly decreased to a normal level (0.109 ± 0.050, mean OD value and SD of the healthy control group). Such decreasing OD value to a normal level indicated successful ABZ or IVM treatment.

### Preparation of GsL3 antigens

GsL3 was obtained from the livers of naturally infected swamp eels purchased from a market in Bangkok. Larvae were used in preparations of three kinds of antigens: crude somatic antigen (CSAg), partially purified antigens (P1Ag, P2Ag and P3Ag) and excretory‐secretory antigen (ESAg). CSAg was prepared by grinding larvae with alumina powder in distilled water, then sonicating and finally obtaining the antigen by centrifugation [5]. The partially purified antigens were prepared by Sephacryl S‐200 gel filtration chromatography and a fraction collector (FRAC‐200; Pharmacia LKB, Uppsala, Sweden). One chromatographic peak was established by pooling fraction tubes, dialysed and concentrated using an Amicon‐stirred ultrafiltration cell at the cut‐off range of 10 kDa (PM10; Ultrafiltration Membrane, Millipore, USA) and the concentrated antigens were called peakAgs: P1Ag, P2Ag and P3Ag [10]. The protein concentration was determined by Coomassie® Plus Protein Assay (Thermo Fisher Scientific, Rock‐ford, IL, USA).

Excretory‐secretory antigen was prepared as previously reported with some modifications [11]. Briefly, 50 GsL3 per ml were cultivated in RPMI‐1640 medium supplemented with 10 mM HEPES, 2 mM glutamine, 100 units/ml penicillin and 100 μg/ml streptomycin in a six‐well culture plate at 37°C and 5% CO_2_ for 6 days. The medium was collected, changed to a new medium every 24 h, pooled, dialysed against ddH_2_O and subsequently concentrated by Amicon at the cut‐off range of 5 kDa (PM‐5 membrane). The protein concentration of ES was determined as mentioned earlier.

### Indirect ELISA

Indirect ELISA was employed to evaluate Ig isotypes and IgG subclasses against various antigens as well as follow‐up analysis after anthelminthic treatments. The ELISA was performed as described previously with some modifications [12]. After checkerboard titrations, each diluted Ag in carbonate‐bicarbonate buffer, pH 9.6, was coated into a Nunc 96‐well microtitre plate (Thermo Fisher Scientific, Rockford, IL, USA) at 37°C and overnight at 4°C, followed by blocking non‐specific binding with 1% skim milk. For antibody‐antigen reaction, diluted serum was added in duplicate wells and incubated, followed by adding horseradish peroxidase (HRP)‐conjugated anti‐human Ig isotype and ‐IgG subclasses (goat anti‐human IgG: KPL, USA; mouse anti‐human IgE, IgM, IgG1, IgG2, IgG3 and IgG4: Southern Biotech, Birmingham, AL, USA). The colorimetric reaction was developed by ABTS (Sigma, Ontario, Canada), and the reaction was stopped with 1% SDS, and OD values were measured at OD 405 nm using an ELISA reader (TECAN, Männedorf, Switzerland).

The best sensitivity and specificity of the ELISA were used in determining the antibody pattern of post‐treated patients every 3 months of the follow‐up period.

### Data analysis

Thirty gnathostomiasis sera and 209 heterologous sera (30 of healthy controls and 179 from infected patients with other parasites) were used for receiver operating characteristic (ROC) curve analysis. The ELISA cut‐off point was chosen using ROC curve analysis as sensitivity versus 1‐specificity or false positive. SPSS version 15 was used as a tool for ROC curve analysis [13]. The accuracy of the diagnostic test was calculated using the method of Galen [14] with GraphPad Prism 5.

## RESULTS

### SDS‐PAGE of various GsL3 antigens

GsL3 antigens; CSAg, ESAg, P1Ag, P2Ag and P3Ag, were analysed for their protein qualities and patterns by SDS‐PAGE before analysis by ELISA (Figure S1). For studying the protein components, 1 µg of each Ag was separated by reducing condition of SDS‐PAGE (ATTO apparatus, Tokyo, Japan), consisting of 5% stacking and 13% separating slab gels and stained with ProteoSilver™ Silver solution. The results showed that CSAg, ESAg and P2Ag exhibited similar banding patterns ranging between 65.71 and 14.4 kDa. P1Ag had banding patterns in the ranging, with an intense band at 32.59 kDa. P3Ag showed an intense band at 24 kDa.

### Optimisation of ELISA conditions

The appropriate conditions were obtained from checkerboard titration: IgM‐ELISA, (1) 0.125 µg/ml CSAg, 1:1600 human serum and 1:8000 HRP‐conjugated mouse anti‐human IgM; and (2) 0.5 µg/ml of each of ESAg, P2Ag and P3Ag, 1:1600 serum, and 1:4000 of such conjugate. For IgG‐, IgG1‐ and IgG4‐ELISAs, their conditions were as follows: (1) 1 µg/ml CSAg, 1:800 serum and 1:8000 HRP‐conjugated mouse anti‐human IgG, ‐IgG1 and ‐IgG4; and (2) for ESAg, P2Ag and P3Ag, 0.5, 4 and 4 µg/ml, respectively, 1:800 serum, and 1:4000 of such conjugates. The optimal conditions of IgE‐, IgG2‐ and IgG3‐ELISAs using all antigens, CSAg, ESAg, P1Ag, P2Ag and P3Ag could not discriminate between pooled positive and pooled negative sera. Therefore, these tests were not further evaluated. In addition, all Igs with P1Ag showed the same evidence of reactions.

### Evaluation of ELISA

Human gnathostomiasis cases, other parasitoses and healthy control sera were employed to determine the sensitivity, specificity and predictive values of ELISA.

### IgM

**FIGURE 1:**
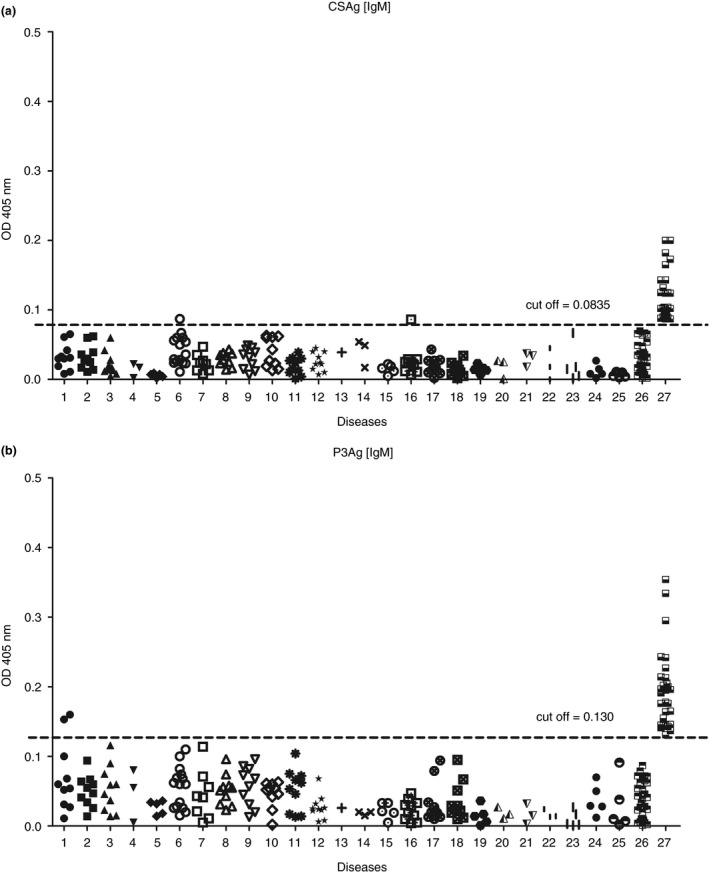
Scatter patterns of OD values of IgM‐ELISA against CSAg (a) and P3Ag (b). Sera associated with several parasitic infections and healthy control sera were analysed, including: 1, strongyloidiasis (10); 2, hookworm infection (10); 3, trichinellosis (10); 4, capillariasis (3); 5, toxocariasis (5); 6, angiostrongyliasis (15); 7, ascariasis (8); 8, trichuriasis (10); 9, bancroftian filariasis (10); 10, brugian filariasis (10); 11, neurocysticercosis (10); 12, taeniasis (10); 13, enterobiasis (1); 14, sparganosis (3); 15, hymenolepiasis nana (5); 16, paragonimiasis heterotremus (10); 17, opisthorchiasis (10); 18, minute intestinal fluke infections (10); 19, fascioliasis (5); 20, creeping eruption (3); 21, entamoebiasis (3); 22, giardiasis (3); 23, *Blastocystis homonis* infection (5); 24, toxoplasmosis (5); 25, malarial infection (5); 26, healthy control (30) and 27, gnathostomiasis (30). Numbers of cases are in parentheses

All antigens to IgM‐ELISAs presented high sensitivity of 100% and high specificity in the range of 91%–99%, with CSAg and P3Ag presenting 99% specificity following cut‐off values of 0.0835 and 0.130, respectively. The OD values of gnathostomiasis cases to CSAg were not far from each other and in the range of 0.086–0.200, while those values of this group to P3Ag were spread across a wider range of 0.131–0.354, with OD values of three cases being outliers. For angiostrongyliasis and paragonimiasis cases, two false positives were using CSAg, giving OD values slightly over the cut‐off value, while P3Ag showed two false positives for strongyloidiasis (Figure 1a and b).

### IgG

IgG‐ELISA against P2Ag demonstrated the highest sensitivity and specificity of 100% and 92.3% respectively (Table 2). The specificities of CSAg and P3Ag were low (80.9% and 83.7%), although their sensitivities were 100%. Meanwhile, ESAg showed the lowest sensitivity (73.3%) and specificity (76.7%).

**TABLE 2:** Demonstration of cut‐off values, sensitivities, specificities and positive and negative predictive values of indirect ELISA against various antigens specific to different Ig isotypes and IgG subclasses

ELISA	Antigen	Cut‐off	Sensitivity (%)	Specificity (%)	Predictive value (%)	
					Positive	Negative
IgM	CSAg	0.0835	100	99	93.75	100
	ESAg	0.027	100	91.90	63.80	100
	P2Ag	0.122	100	94.30	73.17	100
	P3Ag	0.130	100	99	93.75	100
IgG	CSAg	1.046	100	83.70	46.88	100
	ESAg	0.632	100	51.90	22.90	100
	P2Ag	1.042	100	92.30	48.38	100
	P3Ag	0.550	100	80.90	58.33	100
IgG1	CSAg	0.210	100	96.20	78.95	100
	ESAg	0.137	100	95.20	76.92	100
	P2Ag	0.283	100	100	100	100
	P3Ag	0.314	100	100	100	100
IgG4	CSAg	0.442	100	83.30	50.85	100
	ESAg	0.080	100	44.76	20.55	100
	P2Ag	0.189	100	61.70	28.57	100
	P3Ag	0.153	100	90	58.82	100

### IgG subclasses

**FIGURE 2:**
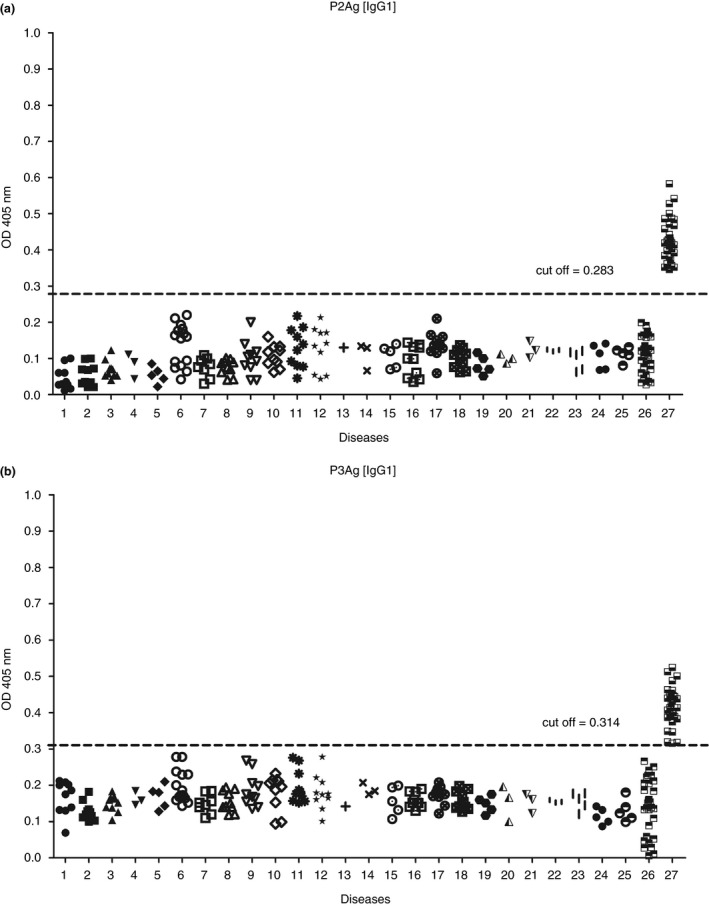
Scatter patterns of OD values of IgG1‐ELISA against P2Ag (a) and P3Ag (b). Sera associated with several parasitic infections and healthy control sera were analysed, including: 1, strongyloidiasis (10); 2, hookworm infection (10); 3, trichinellosis (10); 4, capillariasis (3); 5, toxocariasis (5); 6, angiostrongyliasis (15); 7, ascariasis (8); 8, trichuriasis (10); 9, bancroftian filariasis (10); 10, brugian filariasis (10); 11, neurocysticercosis (10); 12, taeniasis (10); 13, enterobiasis (1); 14, sparganosis (3); 15, hymenolepiasis nana (5); 16, paragonimiasis heterotremus (10); 17, opisthorchiasis (10); 18, minute intestinal fluke infections (10); 19, fascioliasis (5); 20, creeping eruption (3); 21, entamoebiasis (3); 22, giardiasis (3); 23, *Blastocystis homonis* infection (5); 24, toxoplasmosis (5); 25, malarial infection (5); 26, healthy control (30) and 27, gnathostomiasis (30). Numbers of cases are in parentheses

IgG1‐ELISA showed promising results against all antigens. The sensitivity of all tests reached 100%, and the specificity was in the range of 95%–100%. Tests using P2Ag and P3Ag demonstrated the highest specificity of 100% at the cut‐off values of 0.283 and 0.314 respectively. The OD values of gnathostomiasis cases to P2Ag and P3Ag were similar and spread in the ranges of 0.316–0.525 and 0.346–0.583 respectively. P2Ag demonstrated higher specificity to antibodies of gnathostomiasis group than P3Ag because the lowest OD value to P2Ag was far from the cut‐off value but the lowest value to P3Ag was close to its cut‐off value. In addition, IgG1‐P2Ag showed better separation between groups of gnathostomiasis and other infections than IgG1‐P3Ag (Figure 2a and b). Meanwhile, in IgG4‐ELISA, only P3Ag demonstrated the highest specificity of 90% at the cut‐off value of 0.153 (Table 2).

### Post‐treatment antibody levels of gnathostomiasis patients

**FIGURE 3:**
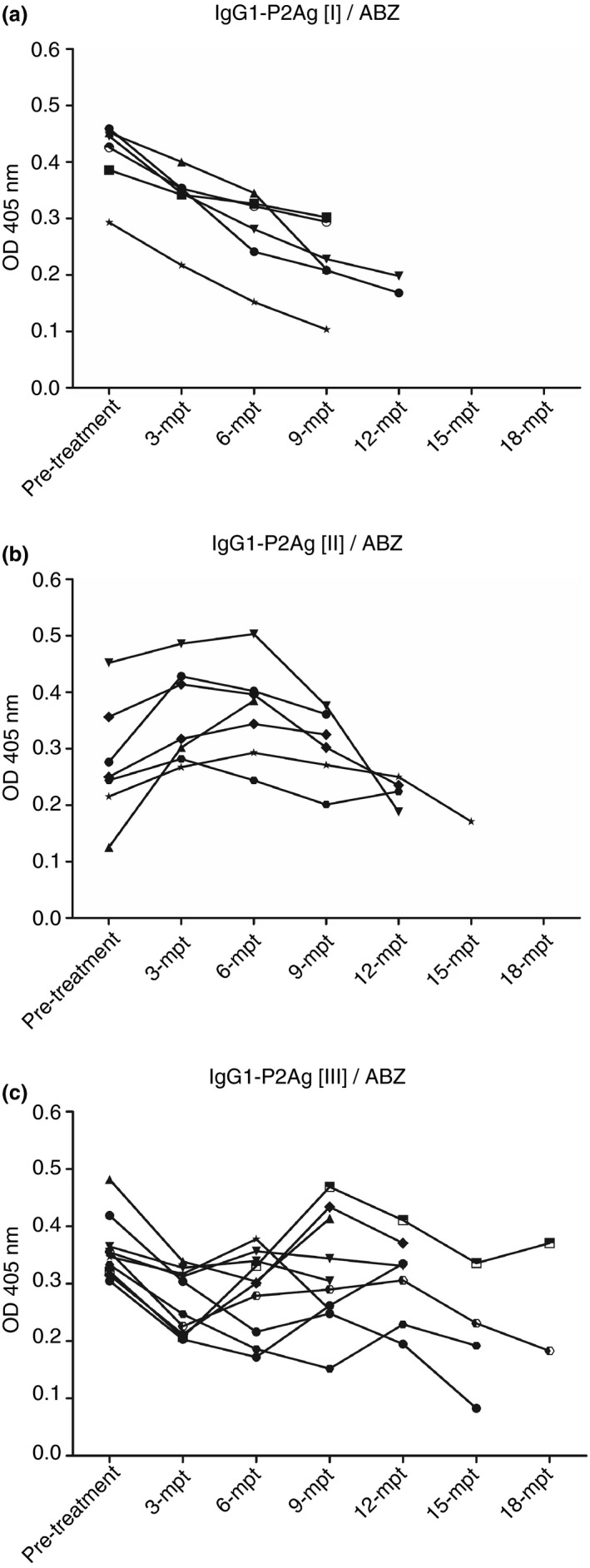
Three patterns of ABZ treatment of human gnathostomiasis using IgG1–ELISA against P2Ag, called P2Ag [I]/ABZ (a), P2Ag[II]/ABZ (b) and P2Ag [III]/ABZ (c). Sera of patients were collected at pre‐treatment and every 3 months post‐treatment (mpt) to 18 mpt with 400 mg of albendazole twice daily for 21 days

**FIGURE 4:**
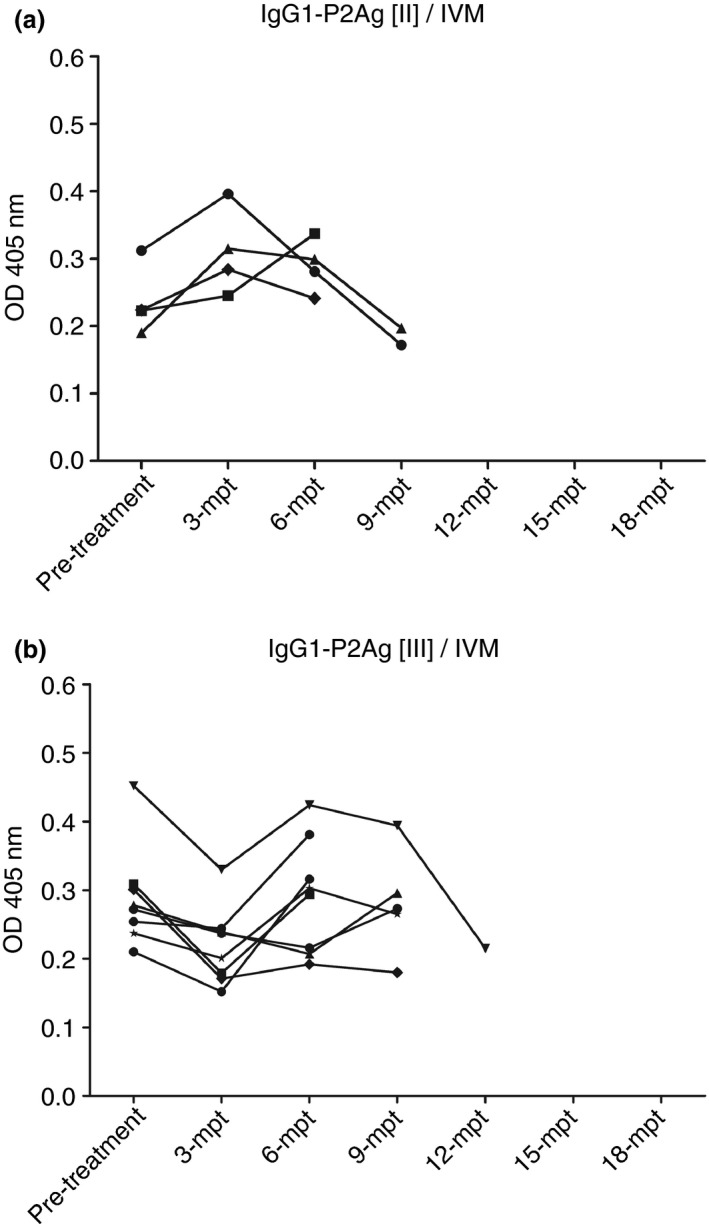
Two patterns of IVM treatment of human gnathostomiasis using IgG1–ELISA against P2Ag, called P2Ag[II]/IVM (a) and P2Ag [III]/IVM (b). Sera of patients were collected at pre‐treatment and every 3 months post‐treatment (mpt) to 18 mpt with 0.2 mg/kg ivermectin (IVM) as a single dose

The highest results of IgG1‐ELISAs were demonstrated as 100% sensitivity and specificity to P2Ag and P3Ag that P2Ag showed better results than P3Ag as mentioned above. Therefore, IgG1‐P2Ag was used to observe the post‐treatment antibody levels of gnathostomiasis sera with ABZ or IVM every 3 months for at least 6 months of antibody observation. A series of serum samples of 23 ABZ‐treated patients demonstrated their antibodies pre‐ and post‐treatment. In IgG1‐P2Ag pattern I (IgG1‐P2Ag [I])/ABZ, six cases demonstrated decreased antibodies from the first 3‐month observation to at least 9 months of follow‐up. These cases appeared to have been cured of gnathostomiasis (Figure 3a). Seven cases in IgG1‐P2Ag [II]/ABZ showed increases and decreases of antibodies during at least 9 months of observation, but only one case showed an antibody increase to 6‐month follow‐up, and the patient did not come to the hospital. However, the antibody of this case might have decreased at the subsequent follow‐up because antibodies of other cases tended to decrease at Months 3–6 (Figure 3b). Ten treated cases had antibodies decreasing and increasing for at least 12 months in the pattern of IgG1‐P2Ag [III]/ABZ. Several cases showed antibody levels over 18 months, and only 2−3 showed antibodies decreasing (Figure 3c). For IVM treatment (Figure 3a), antibodies of four cases of IgG1‐P2Ag [II]/IVM showed increases and decreases; two cases at least 6 months came to follow‐up process and antibodies of another two cases showed antibodies going down at 9 months of the follow‐up (Figure 4a). Eight cases of IgG1‐P2Ag [III]/IVM pattern had antibodies decreasing and increasing, and the antibody of one case decreased at 9‐month observation (Figure 4b). There was no pattern [I] of IVM treatment as that of the pattern [I] of ABZ treatment. Relapse occurred in both ABZ and IVM treatments, as shown by the patterns of OD values [III] in follow‐up observation. In the study, several patterns of antibody levels were found in both treatments and could be divided into three main patterns as follows.

I: Gradually decreased values from the onset of infection: The declining OD values might be interpreted as patients having been cured of parasitic infection by ABZ or IVM.
II: Fluctuating values with antibodies decreasing at the end: Antibody levels demonstrated increasing OD values at the onset of infection, and antibody levels would decrease, increase and finally decrease at the end of ABZ or IVM observation. This occurrence indicated that the worm was still alive but might not have been active or almost dead.
III: Somewhat steady values, with antibody levels down at the onset of infection, then regularly elevated and declined. The worm was alive and would survive for an extensive follow‐up.

In the history of treatments, one *Gnathostoma* worm was extracted from each of the patients treated with ABZ or IVM.

## DISCUSSION

### Non‐specificity of immunoglobulins to antigens in ELISA

Immunodiagnoses of gnathostomiasis have been consecutively developed to improve their efficiency. Several strategies to improve the reliability of diagnosis suggested promising results based on the evaluation of immunoglobulins specific to *G*. *spinigerum* infection, use of various types of Ag and infections with a large number of different parasites [4, 5, 8, 15, 16, 17, 18 ].

In our study, the checkerboard titrations of IgE‐, IgG2‐ and IgG3‐ELISAs using CSAg, ESAg, P1Ag, P2Ag and P3Ag, could not discriminate between pooled positive and pooled negative sera. Those Ags might be non‐immunogenic components to the positive controls; therefore, the detection of IgE, IgG2 and IgG3 was not continued. This study attempted to conclude 25 parasitic infections with many samples (n = 179) to achieve the best specificity of tests. For IgE‐ELISA, the low specificity presented in this study was consistent with a previous study showing low sensitivity and specificity against CSAg and ES of GsL3 [19]. Moreover, another study determining the total IgE level in gnathostomiasis cases compared with the controls (other parasitic infections, allergy and autoimmune diseases) found no statistically significant difference between them [20]. A low level in gnathostomiasis cases may indicate an insufficient amount of gnathostomiasis‐specific IgE to differentiate between infected and control sera. Two gnathostomiasis cases were proven by visiting an endemic area, consuming raw fish, clinical symptoms and ELISA, but those cases had normal IgE levels [21]. These findings also support our study on the lack of discrimination of OD values between cases of gnathostomiasis and healthy controls in checkerboard titration.

### Specificity of immunoglobulins to antigens in ELISA

IgM‐ELISAs demonstrated a sensitivity of 100% for all Ags, along with a remarkable specificity of 91.9%–99%, while CSAg and P3Ag showed 99% specificity. A study on IgM for gnathostomiasis is uncommon, but our work demonstrated the excellent specificity for CSAg and P3Ag. Regarding the timeline of the responsive antibody, IgM rapidly responds and can be detected at an early stage during the first 2 weeks, followed by IgG antibody [22, 23]. IgG slowly increases and can be tested for long, making it appropriate for analysing follow‐up treatment patients. Therefore, IgM‐ELISA with CSAg and P3Ag may be useful for the early diagnosis of human gnathostomiasis. However, acute‐phase sera and a more significant number of gnathostomiasis cases with different clinical manifestations need to be recruited to confirm this. In a gnathostomiasis case returning from Mexico, three normal IgM level results were shown, so it seems that IgM did not respond to the parasite in that case [24].

IgG‐ELISA against CSAg and ESAg in our study presented low specificity, which was consistent with a previous study that showed high cross‐reaction of heterologous infections using CSAg [4]. However, our specificity (83.7%) was far higher than their study (8%). These different specificities could be due to the IgG‐ELISA conditions in each laboratory. At 100% sensitivity, P2Ag showed higher specificity (92.3%) than CSAg (83.7%), ESAg (51.9%) and P3Ag (80.9%). Our results of IgG‐ELISA using P2Ag were similar to those of using 136 sera associated with infections by 17 different parasites (100% sensitivity and 91.3% specificity) using C1‐Ag (purified by SephadexG‐200 column chromatography), except for D1‐Ag (purified by DE‐52, anion exchange column chromatography) which had 100% sensitivity and 100% specificity in their study [8]. For a partially purified antigen, eluted Ag containing low‐molecular‐weight components (<27 kDa), IgG‐ELISA gave 100% sensitivity, specificity and predictive value, with this specificity obtained from analyses on only seven helminthic infections [17]. For the produced Ag using trichloroacetic acid/acetone precipitation technique, the concentrated Ag was analysed by IgG‐ELISA and separated by OD values between gnathostomiasis and healthy controls. The Ag was further analysed by dot immune‐gold filtration assay (DIGFA) and showed 96.7% sensitivity and 100% specificity. Its specificity was determined using 155 sera associated with six different helminthiases [18]. Our study using P2Ag demonstrated lower specificity (92.3%), but this was determined using 179 sera of infections with 25 parasites.

For IgG subclass‐ELISA, IgG1 was an excellent immunoglobulin to bind on these four Ags at 100% sensitivity and 95%–100% specificity. Using P2Ag and P3Ag, IgG1‐ELISA achieved perfect results of 100% sensitivity and 100% specificity. However, P2Ag can well separate positive gnathostomiasis controls from heterologous sera compared with P3Ag at their cut‐off values (Figure 2a and b). Generally, high cross‐reactivity of CSAg with antibodies associated with other parasitic infections occurs, such as 83.70% and 83.30% specificities of IgG‐ELISA including IgG4‐ELISA, respectively, but IgG1‐ELISA showed 96.2% specificity following its appropriate conditions of checkerboard titration. Therefore, CSAg can possibly be used in IgG1‐ELISA to detect gnathostomiasis because the Ag preparation is simple, quick and low‐cost. Moreover, P2Ag using IgG1‐ELISA is considered to increase the reliability of diagnosis. IgG subclasses and GsL3Ag can be combined; IgG1‐ELISA with 98% sensitivity was used for screening tests, while IgG2‐ELISA with 88% specificity was used for confirming tests [4].

### Evaluation of ELISA for following‐up of gnathostomiasis treated patients

Anthelminthic treatment is a standard method for curing gnathostomiasis, with albendazole (ABZ) or ivermectin (IVM) being the drugs of choice [25]. It is known that ABZ or ivermectin treatment alone might fail and that repeated treatments or a combination of ABZ and ivermectin can finally be successful in gnathostomiasis. However, long‐term administration of ABZ (400 mg twice daily for 21 days) and the efficacy of IVM (76% cure rate) are doubtful to the treatment [26, 27], and the lack of a reliable tool for following up treatment efficacy was raised as the main reasons for treatment failure. Regarding the duration of the follow‐up period, several cases showed long treatment with those drugs, but those drugs cured several cases with/without recurrence. There were also problems with the follow‐up visits, in that patients frequently missed appointments because of no recurrence or symptoms, their occupation, residing far from the hospital and other factors. Finally, it is possible to observe long‐term treatment, which may depend on the efficiency of anti‐helminthic drugs, the pharmacokinetics of drugs in patients or anything affecting drug function. For example, different hours of ABZ metabolites by pharmacokinetic analysis were found in serum and urine samples of individual volunteers [28]. This may affect parasites to anti‐helminthic drugs in short or long exposure time. In the ABZ follow‐up observation, relapse occurred as shown in the pattern of OD values [III], which was the same as in studies on gnathostomiasis by Strady et al. [27] and Ménard et al. [29]. However, this did not occur in ABZ treatment in a study by Kraivichian and colleagues [26].

Regarding the data above [21, 27, 29], in studies with a long follow‐up with ABZ and IVM, 12–24 months may be reached with no recurrence. Therefore, the result of treatment should continue in the long term. Evaluation of drug treatment's efficacy in humans has relied mostly on clinical signs and symptoms, such as the disappearance of swelling during the follow‐up period. In addition, a decline of antibody levels in humans is also one of the criteria indicating curative treatment.

## CONCLUSION

IgM and IgG1 were shown to be optimal for human gnathostomiasis diagnosis using CSAg, P2Ag and P3Ag (100% sensitivity and >99% specificity). The use of IgG1‐ELISA with P2Ag and P3Ag achieved perfect results of 100% sensitivity and 100% specificity. These tests can be an alternative method for the immunoblotting test of human gnathostomiasis. Screening and early detection tests require CSAg and P3Ag to IgM (100% sensitivity and 99% specificity). Therefore, the follow‐up duration should be at least 12 months.

## CONFLICTS OF INTEREST

The authors declare that they have no competing interests.

## Supporting information

Figure S1
