# Supplementary figures and images for: Evaluation of immunodiagnostic tests for human gnathostomiasis using different antigen preparations of Gnathostoma spinigerum larvae against IgE, IgM, IgG, IgG1‐4 and IgG1 patterns of post‐treated patients
Source: Trop Med Int Health. 2021 Sep 21;26(12):1634–44. doi: 10.1111/tmi.13679 (PMC9291276; doi:10.1111/tmi.13679)

**Supporting Information**

**
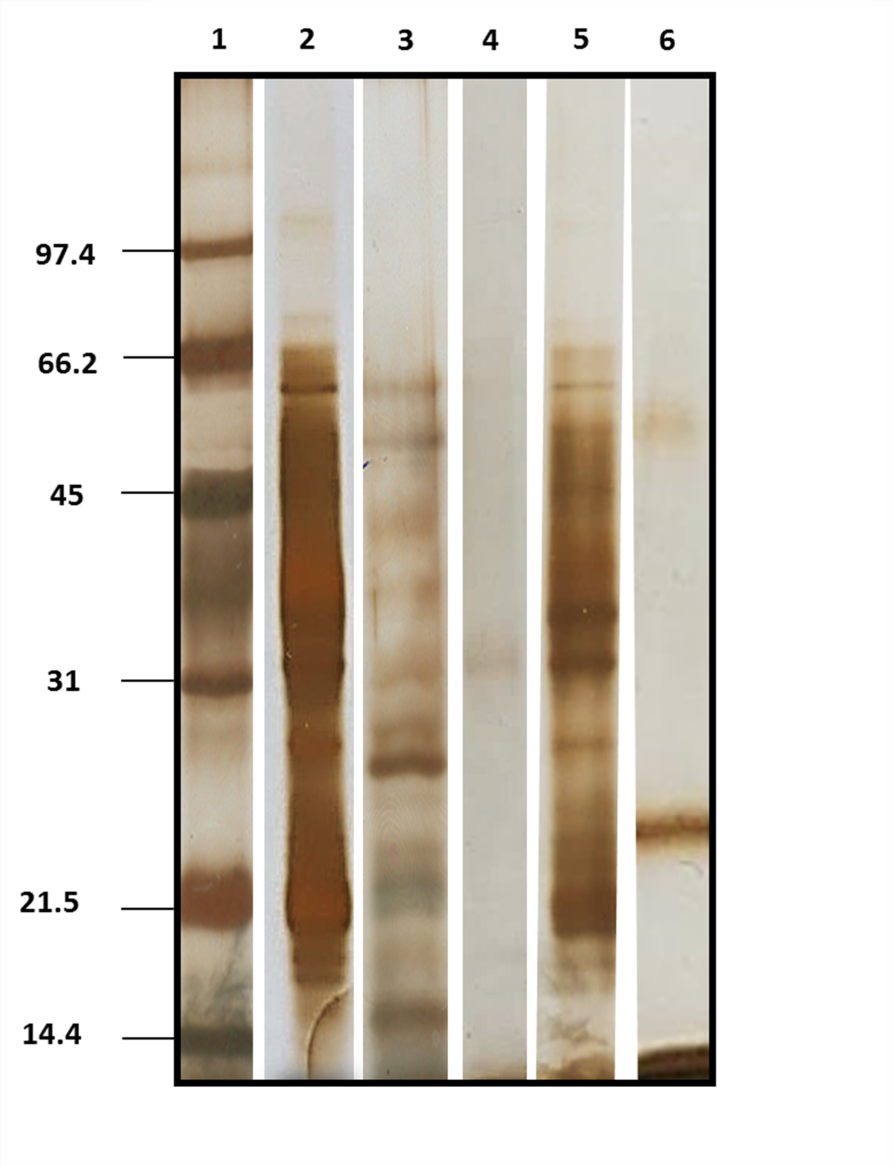
**

**Figure S1**

Supplement: Supplementary file 1 — Figure S1 [file TMI-26-1634-s001.docx]
